# The effect of menopause and hysterectomy on systemic vascular endothelial growth factor in women undergoing surgery for breast cancer

**DOI:** 10.1186/1471-2407-8-279

**Published:** 2008-09-30

**Authors:** Aoife J Lowery, Karl J Sweeney, Alan P Molloy, Emer Hennessy, Catherine Curran, Michael J Kerin

**Affiliations:** 1 Department of Surgery, Clinical Science Institute, University College Hospital Galway, Galway, Ireland

## Abstract

**Background:**

Vascular endothelial growth factor (VEGF) is a potent angiogenic cytokine produced physiologically by the uterus. Pathological secretion by tumours promotes growth and metastasis. High circulating VEGF levels potentially have a deleterious effect on breast cancer by promoting disease progression.

The aims of this study were to investigate circulating VEGF levels in breast cancer patients and assess the effect of menopause or hysterectomy on systemic VEGF.

**Methods:**

Patients undergoing primary surgery for breast cancer and controls matched for age, menopausal and hysterectomy status were prospectively recruited. Serum VEGF, FSH, LH, estrogen, progesterone and platelet levels were measured. Serum VEGF was corrected for platelet load (sVEGFp) to provide a biologically relevant measurement of circulating VEGF. SVEGFp levels were analyzed with respect to tumor characteristics, menopausal status and hysterectomy status.

**Results:**

Two hundred women were included in the study; 89 breast cancer patients and 111 controls. SVEGFp levels were significantly higher in breast cancer patients compared to controls (*p *= 0.0001), but were not associated with clinico-pathological tumor characteristics. Systemic VEGF levels reduced significantly in the breast cancer patients following tumor excision (*p *= 0.018). The highest systemic VEGF levels were observed in postmenopausal breast cancer patients. Postmenopausal women who had had a previous hysterectomy had significantly higher VEGF levels than those with an intact postmenopausal uterus (p = 0.001).

**Conclusion:**

This study identifies an intact postmenopausal uterus as a potential means of reducing circulating levels of VEGF which could confer a protective effect against breast cancer metastatic potential.

## Background

Vascular Endothelial Growth Factor (VEGF) is one of the most potent angiogenic cytokines, acting as a specific endothelial mitogen capable of stimulating angiogenesis in vitro and in vivo [1].

The adult female reproductive tract, under ovarian steroid hormone influence, is the principle endogenous VEGF producer and the only site where physiological angiogenesis occurs routinely. VEGF ligands and receptors are expressed in the uterine endometrium [2] and myometrium [3] and cyclical variations in serum VEGF during the menstrual cycle have previously been demonstrated [4,5].

VEGF is also a predominant growth factor involved in the pathological angiogenesis that drives cancer progression and spread [6] and serum VEGF is elevated in patients with different tumor types, including breast cancer [7,8].

The ovarian steroid hormones estrogen and progesterone play an important role in breast cancer development and have been shown to influence VEGF expression in breast cancer and non-cancer tissue, thus contributing to tumour-derived and tumour independent circulating VEGF levels [9-13]. Clinical studies have provided evidence for a link between hormone-dependent breast cancer and the VEGF pathway [14-16]

The postmenopausal change in ovarian sex hormones would be expected to alter VEGF production and subsequent release in the reproductive tract, and we would expect a consequent postmenopausal change in systemic VEGF levels. However, studies to date on the effect of menopause on circulating VEGF and breast tumoural VEGF have been equivocal; with conflicting reports of increased VEGF levels in premenopausal [17] and in postmenopausal women[18,19], or no difference in VEGF levels according to menopausal status [20]. Furthermore, as the uterus is an endogenous VEGF source, we hypothesize that hysterectomy would also effect VEGF levels. An alteration in VEGF synthesis and subsequent release would potentially influence breast cancer cell dissemination and response to therapy in breast cancer patients.

Our aims in this study were to investigate circulating VEGF levels in breast cancer patients and assess the effect of menopause or hysterectomy on systemic VEGF.

## Methods

A consecutive series of patients undergoing curative surgery for breast cancer over a six month period at a single institution were invited to participate in the study. Informed consent was obtained as approved by the University College Hospital Galway ethics committee. Breast cancer patients underwent radiological investigation and had a histological diagnosis by core biopsy. Preoperative chest x-ray, full blood count, serum electrolytes and liver function tests were performed in all patients. Computerized tomography of chest thorax and abdomen and isotope bone scan was performed selectively. No patient received neoadjuvant cytotoxic or hormonal chemotherapy. All surgery was performed by one of two consultant surgeons.

A cohort of healthy, non-surgical, female volunteers was recruited to the study during the same time period. These women were recruited in the general surgical outpatient clinic and had no history of malignancy or recent surgery. At the time of recruitment all participants completed a detailed questionnaire regarding their medical, surgical and gynecological history. Women with a history of renal failure, diabetes mellitus, lung disease or infectious disease were excluded from the study

Menopausal status at recruitment was defined by each woman's reported menstrual history and ovarian function tests. Postmenopausal women had experienced no menstrual period for at least 12 months, had a previous bilateral oopherectomy, or had postmenopausal levels of FSH (follicle stimulating hormone), lutenizing hormone (LH) and oestradiol. Premenopausal women were experiencing a normal menstrual cycle. Patients who had previously undergone a hysterectomy or were currently taking postmenopausal hormone therapy were categorized according to the Million Women Study protocol [21].

Peripheral venous blood samples were obtained prior to induction of anaesthesia and at least six weeks after definitive surgery. All postoperative samples were taken before commencement of adjuvant systemic cytotoxic chemotherapy or radiotherapy and in the absence of residual or metastatic disease in the study group and at the time of recruitment in the control group.

Samples for VEGF analysis were collected in Vacutainer SST II (serum separator tubes), transported on ice and allowed to clot for 1 hour prior to centrifugation at 3000 r.p.m. at 4°C for 10 minutes. Serum was then separated, aliquoted into 2 ml tubes and stored at -20°C.

Serum VEGF (sVEGF) levels were determined using a commercially available quantitative sandwich immunoassay technique (Quantikine; R&D System, Minneapolis, MN, USA). VEGF_165 _the predominant and most biologically active isoform was measured in this study. Briefly, a monoclonal antibody specific for VEGF_165_, pre-coated onto a micro-titre plate, was used to capture the VEGF from serum samples. An enzyme linked polyclonal antibody specific for VEGF was added, followed by a substrate solution and used for quantification. All samples were tested in duplicate. The minimal detectable dose of VEGF was 9.0 pg/ml and maximum dose was 2000 pg/ml. For serum samples the intra-assay variation of the assay kit used was 4.5–6.7% and the inter-assay variation was 6.2–8.8%.

To correct for variation in platelet counts between patients, sVEGF levels were corrected for platelet load according to the equation sVEGF(picogram/millilitre (pg/ml)) ÷ platelet count × 10^6^/ml = sVEGFp (pg/10^6^) [22]

Serum levels of FSH, LH, estradiol and progesterone were determined using a commercially available chemiluminescence assay system (ADVIA Centaur^®^, Bayer Diagnostics, Tarreytown, NY, USA)

Statistical analysis was performed using SPSS version 14.0 software. Results are presented as median and interquartile range (IQR) unless otherwise stated. The differences in sVEGFp levels were analysed using Kruskal-Wallis or Mann Whitney tests to compare between groups. The Wilcoxin signed rank test was used when examining for significant changes. Mann Whitney and Spearman's correlation tests were used as appropriate to investigate relationships between sVEGFp and other clinicopathological variables. P < 0.05 was considered statistically significant.

## Results

Two hundred women were recruited to the study: 89 women who underwent curative breast cancer surgery (study group) and 111 women with no history of malignancy or recent surgery (control group).

The tumor characteristics of the breast cancer patients are illustrated in table 1.

**Table 1:** Tumour Characteristics

**Tumour Characteristic**	**All Breast Cancers** **N = 89**	**Premenopausal Breast Cancer** **N = 25**	**Postmenopausal Breast Cancer** **N = 64**	**p-value**
**Histologic Subtype**				
-Invasive Ductal (%)	67(75)	19(76)	48(75)	
-Invasive Lobular (%)	12(13.5)	3(12)	9(14)	0.1
-DCIS (%)	4(4.5)	0	4(6)	
-Other (%)	6(7)	3(12)	3(5)	

Median tumor size (IQR) mm	25 (16–35)	28 (15–36)	24.5 (16.25–35)	0.955

Lymph node +ve (n)(%)	49 (55)	16(66)	33(52)	0.347

**Receptor Status**				
-ER Positive (%)	78 (88)	24(96)	54(84)	0.056
-PR Positive (%)	81(91)	24(96)	57(89)	0.117
-Her2neu Positive (%)	15(17)	3(12)	12(19)	0.47

**Tumor Grade**				
-Grade 1 (%)	19(21)	8(32)	11(17)	
-Grade 2 (%)	43(48)	10(40)	33(52)	0.183
-Grade 3 (%)	27(31)	7(28)	20(31)	

**UICC Stage**				
-Stage 0 in-situ (%)	4(4)	0	4(6)	
-Stage 1 (%)	19(21)	5(20)	14(22)	
-Stage 2a (%)	21(24)	7(28)	14(22)	0.657
-Stage 2b (%)	26(29)	9(36)	17(27)	
-Stage 3a (%)	13(15)	2(8)	11(17)	
-Stage 3b (%)	6(7)	2(8)	4(6)	

Among the study group there were 40 breast-conserving procedures and 49 mastectomies performed. All procedures were performed under general anaesthetic and all patients undergoing immediate breast reconstruction (n = 14) had an additional paravertebral anaesthetic block. 58 patients had an axillary clearance and 30 had sentinel lymph node biopsy only. One patient, with low grade ductal carcinoma in situ (DCIS) did not have axillary nodal evaluation.

Serum VEGF was elevated in the study group (table 2). Six weeks following tumor resection, these levels dropped significantly, although they remained elevated compared to the control group. (median pre-operative sVEGFp (IQR) vs median post-operative sVEGFp (IQR): 1.08(0.68–1.44) pg/10^6 ^vs 0.97(0.58–1.3) pg/10^6 ^respectively; p = 0.018)

**Table 2:** Baseline Patient Characteristics, Serum Steroid Hormone and VEGF levels

**Group**	**Control (n = 111)**		**Breast Cancer (n = 89)**	
Median age (IQR) yrs	53 (41–61)		57 (50–67)	

Menopausal Status (n)	Premenopausal (43)	Postmenopausal (68)	Premenopausal (25)	Postmenopausal (64)

Median Oestrogen (IQR) pmol/l	415.5(251.5–645.2)	<100	491.0(267.2–572.7)	<100

Median Progesterone (IQR) nmol/l	2.5 (1.45–18.7)	1.00 (0.8–1.1)	4.7 (3.1–14.7)	0.8 (0.67–1.47)

Median sVEGF (IQR) pg/ml	117.12 (73.36–295.58)		306.67 (176.2–407.7)*	
	
	143.3(56.4–240.4)	177.8(91.9–312.9)	225.1(98.2–344.4)	326.0**†**(206.0–420.3)

Median sVEGFp (IQR) pg/10^6^	0.73 (0.34–1.24)		1.08 (0.68–1.44)*	
	
	0.58 (0.26–1.16)	0.75(0.41–1.30)	0.77 (0.31–1.4)	1.11 **†**(0.80–1.47)

*p = 0.0001 vs Control Group levels

**† **p < 0.05 vs Premenopausal study group & Pre- & Postmenopausal Control Group levels

In the premenopausal women, there was no difference in sVEGFp levels between the study and control groups; the highest sVEGFp levels were observed in the postmenopausal breast cancer patients (Table 2).

Within the study group, sVEGF and sVEGFp were elevated in the postmenopausal patients compared to premenopausal patients.

This difference was not observed in the control group (Table 2).

Twenty-seven (42%) of the 64 postmenopausal study patients and 32 (47%) of the 68 postmenopausal controls had previously had a hysterectomy for benign disease.

**Figure 1:**
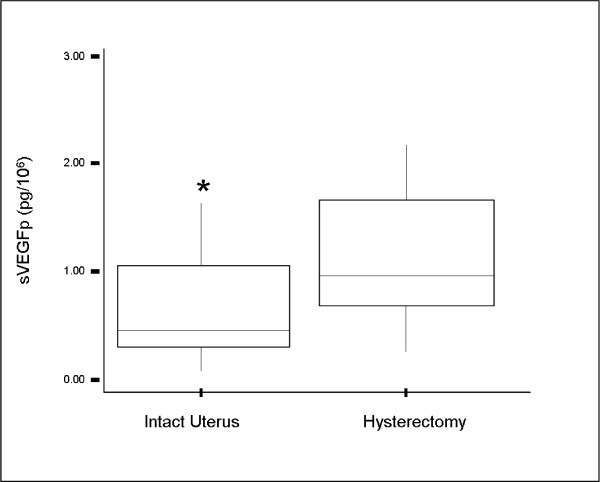
**sVEGFp levels in postmenopausal controls (n = 68)**:  there was a significantly higher sVEGFp level in women who had a previous hysterectomy (n = 32) compared to those with an intact postmenopausal uterus (n = 36).  * p = 0.001.

Postmenopausal controls who previously had a hysterectomy had higher sVEGFp levels than those with an intact uterus (median sVEGFp (IQR) hysterectomy vs intact uterus: 1.01(0.72–1.66) pg/10^6 ^vs 0.50(0.3–1.07) pg/10^6 ^respectively; p = 0.001) (figure 1)

This difference was not seen in the study group (median sVEGFp (IQR) hysterectomy vs intact uterus 1.11(0.95–1.28) pg/10^6 ^vs 1.11(0.77–1.55) pg/10^6 ^respectively; p = 0.43).

**Figure 2:**
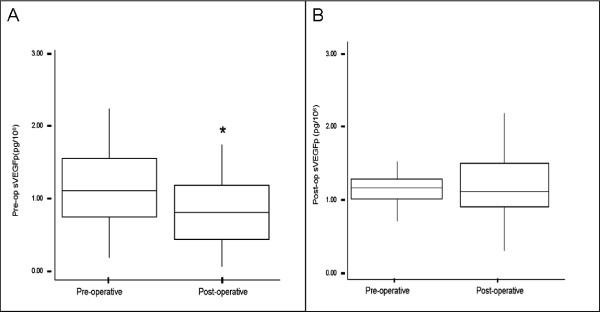
**sVEGFp levels in postmenopausal breast cancer patients (n = 64)**. **2a: **sVEGFp levels decreased significantly following tumour excision in postmenopausal breast cancer patients with an intact uterus (n = 37). *p = 0.003. **2b: **sVEGFp levels did not decrease significantly following tumour excision in postmenopausal breast cancer patients who had previously undergone hysterectomy (n = 27).

Serum VEGFp decreased postoperatively in the postmenopausal study group with an intact uterus (median sVEGFp (IQR) preoperative vs postoperative: 1.11(0.77–1.55) pg/10^6 ^vs 0.95 (0.48–1.24) pg/10^6 ^respectively; p = 0.003). There was no significant post-operative reduction in sVEGFp in patients who had undergone a previous hysterectomy (p = 0.101, figure 2a &2b)

There was no association found between sVEGFp and clinicopathological prognostic parameters including nodal status (p = 0.347), tumor size (p = 0.301) and tumor grade (p = 0.284).

At a median follow up of 28 months there were four disease recurrences; one premenopausal patient who had stage 3 disease at presentation developed distant metastases. Three postmenopausal patients developed recurrence: 1 distant metastasis and 2 contralateral breast cancers. Two of the recurrences occurred in postmenopausal women who had a previous hysterectomy.

## Discussion

Systemic VEGF levels are elevated in breast cancer patients, reflecting tumour derived VEGF. The significant decrease in circulating VEGF levels following primary excision of the tumour supports the hypothesis that tumour derived VEGF contributes to circulating VEGF levels in breast cancer patients.

Tumour derived VEGF drives angiogenesis and enhances the potential for tumour growth, dissemination of malignant cells and metastasis [6]. In breast cancer patients, higher serum VEGF levels have been reported in patients with metastatic disease compared to non-metatstatic breast cancer patients and controls [20]. Serum VEGF has been identified as a useful prognostic marker in malignancy [23-25], however its clinical usefulness remains questionable in breast cancer patients.

The physiological production of VEGF in the reproductive tract contributes to circulating VEGF levels and the ovarian steroid hormones have been shown to influence VEGF expression in both uterine and breast tissue [2,9,11,26]. The cyclical variation in VEGF levels [4,5] is likely to obscure or even eclipse tumor production of VEGF rendering serum VEGF levels unreliable as a disease marker in breast cancer, despite having proven use in predicting prognosis in a number of other cancers [23-25]. We did not observe any significant difference in systemic VEGF levels between premenopausal patients with breast cancer and controls, supporting the evidence that the reproductive tract and hormonal milieu contribute significantly to systemic VEGF levels in premenopausal women. Furthermore, this study did not demonstrate any significant correlation between pre-operative serum VEGF levels and tumor characteristics used in prognostication such as grade, stage and lymph node status in the breast cancer group, this finding is in concordance with others, who have reported limited usefulness of VEGF as a tumour marker in breast cancer [19,27].

Serum VEGF represents both the circulating plasma VEGF and VEGF released from the alpha granules of circulating platelets on coagulation [28,29]. It has been suggested that platelets act as scavengers of circulating and tumour produced VEGF in malignancy [22,30]. For this reason, we have corrected the serum VEGF levels according to the platelet load of the control group and the study group pre- and post-operatively to obtain biologically relevant sVEGFp levels.

When divided on the basis of menopausal status there was a significant difference in circulating VEGF levels between breast cancer patients and controls only in the postmenopausal cohort.

The highest serum VEGF levels were observed in the postmenopausal breast cancer patients. Considering the postmenopausal decrease in ovarian function and production of ovarian steroid hormones it is likely that tumor VEGF production contributes largely to circulating VEGF in this group.

The unexpected finding of higher serum VEGF levels in women who had a previous hysterectomy implies a previously unrecognized influence of the postmenopausal uterus on circulating VEGF levels. This finding is in contrast to previous reports of lower VEGF levels in postmenopausal patients who had undergone hysterectomy [19,31]. Agrawal *et al *[31] reported significantly higher serum VEGF levels in postmenopausal patients with an intact uterus than those who had a previous hysterectomy, if the patients were not using hormone replacement therapy (HRT). However, in their analysis, the authors compared 34 patients with an intact uterus with only 6 patients in the hysterectomy group. Interestingly, when they compared the patients who were taking HRT, in groups with larger numbers, it was found that patients with an intact uterus (n = 98) had lower VEGF levels than those who had a previous hysterectomy (n = 61). This finding was attributed to the HRT preparations being used; the patients with an intact uterus received combined estrogens and progesterones, while those with a hysterectomy received unopposed estrogen. However, they found no difference in serum VEGF levels when comparing the use of these HRT preparations in all patients. Coskun *et al *[32] have reported a reduction in serum VEGF levels in postmenopausal breast cancer patients receiving hormonal therapy in the presence of a normal endometrium, with increased circulating VEGF in patients with endometrial thickening. Taken together these findings suggest that the postmenopausal uterus may indeed play an active role in the regulation of circulating VEGF levels.

The observed reduction in circulating VEGF observed in postmenopausal women with an intact uterus could be mediated via decreased VEGF production or an active role of the uterus in reducing circulating VEGF levels via sequestration or reduction of VEGF bioactivity. The binding of free VEGF to the tyrosine kinase VEGF receptors modulates VEGF bioavailability and may reduce soluble VEGF levels [33]. Alternatively, there may be an active role for the postmenopausal aromatase produced steroid hormones in the regulation of circulating VEGF levels [12,34]. Further study is required to clarify the temporal relationship between hysterectomy and alteration in serum VEGF levels and to elucidate the precise mechanisms by which the postmenopausal uterus may reduce circulating VEGF.

In postmenopausal breast cancer patients, where tumour-derived VEGF was also contributing to circulating VEGF levels, there was no difference in pre-operative VEGF levels between those who had a previous hysterectomy and those who had an intact uterus. The decrease in serum VEGF levels post-operatively, following excision of the tumour, was significantly more marked in the patients with an intact uterus, mirroring the findings in the control group and supporting the hypothesis that an intact postmenopausal uterus has an impact on systemic VEGF levels. In this manner, the presence of an intact uterus may confer a protective effect in malignancy via a reduction in circulating VEGF. A large cohort study addressing the effect of previous hysterectomy on prognosis in breast cancer patients will identify the oncologic significance of these findings. If found to be at an increased risk of breast cancer progression, women who have had a previous hysterectomy may benefit from more rigorous surveillance or targeted therapy

## Conclusion

In conclusion, serum VEGF appears to be influenced by the presence of a postmenopausal uterus. In patients with breast cancer this may represent a mechanism of reducing the levels of circulating VEGF available for pathological tumor angiogenesis, the prognostic significance of this influence warrants further investigation.

## Abbreviations

VEGF: Vascular Endothelial Growth Factor; FSH: Follicle Stimulating Hormone; LH: Lutenizing Hormone; sVEGF: Serum Vascular Endothelial Growth Factor; sVEGFp: Serum Vascular Endothelial Growth Factor corrected for platelet load (serumVEGF sVEGF(pg/ml) ÷ platelet count × 10^6^/ml = sVEGFp (pg/10^6^)^1^; VEGF_165_: Vascular Endothelial Growth Factor isoform 165; IQR: Interquartile Range; DCIS: Ductal Carcinoma in Situ; ER: Estrogen Receptor; PR: Progesterone Receptor.

## Competing interests

The authors declare that they have no competing interests.

## Authors' contributions

AJL participated in study design, selected and recruited patients, collected serum samples, carried out the ELISAs, collated the data, performed statistical analysis and drafted the manuscript. KJS conceived of the study, participated in its design and co-ordination and helped draft and critically review the manuscript. APM participated in study design, patient selection and recruitment and sample acquisition. EH and CC participated in sample collection and preparation, collation of clinical and histological data and statistical analysis. MJK participated in study concept, design and critical review of manuscript.

All authors read and approved the final manuscript.

## Pre-publication history

The pre-publication history for this paper can be accessed here:


